# Risks of Miniplate Removal in Fibula Free Flap Oromandibular Reconstruction

**DOI:** 10.1002/oto2.70214

**Published:** 2026-03-03

**Authors:** Kuo‐Chung Yang, Wen‐Chung Liu

**Affiliations:** ^1^ Department of Surgery, Division of Plastic and Reconstructive Surgery Kaohsiung Veterans General Hospital Kaohsiung Taiwan (R.O.C.); ^2^ Department of Surgery, School of Medicine, College of Medicine National Yang Ming Chiao Tung University Taipei Taiwan (R.O.C.); ^3^ School of Medicine, College of Medicine National Sun Yat‐sen University Kaohsiung Taiwan (R.O.C.); ^4^ Institute for Translational Research in Biomedicine Kaohsiung Chang Gung Memorial Hospital Kaohsiung Taiwan (R.O.C.)

**Keywords:** free fibula flap, miniplate removal, oromandibular reconstruction, osteotomies, radiotherapy, surgical complications

## Abstract

**Objective:**

To identify clinical and surgical risk factors for miniplate removal after oromandibular reconstruction with fibula free flap (FFF) and evaluate the timing of this complication.

**Study Design:**

This was a retrospective cohort study.

**Setting:**

This study was conducted at Kaohsiung Veterans General Hospital, a tertiary care center, from January 2015 to December 2023. Patients undergoing oromandibular reconstruction with FFF were categorized by miniplate removal status.

**Methods:**

The incidence of miniplate removal and postoperative complications were analyzed. Multivariate logistic regression identified independent risk factors, and Kaplan‐Meier analysis assessed the time to miniplate removal.

**Results:**

Out of 184 patients, 30 (16.3%) experienced miniplate issue required removal. This group showed significantly higher complication rates, including abscess/fistula (53.3% vs 5.8%), osteomyelitis (43.3% vs 0.6%), and osteoradionecrosis (23.3% vs 2.6%) (*P* < .001 for all). Preoperative or postoperative radiotherapy (OR, 9.27; *P* = .046) and number of osteotomies (OR, 3.77; *P* = .038) were identified as independent risk factors. Kaplan‐Meier analysis indicated a trend of higher miniplate removal rates in the radiotherapy group (*P* = .055), with the highest risk within the first 30 months.

**Conclusion:**

Radiotherapy and an increased number of osteotomies are independent risk factors for miniplate removal after FFF reconstruction. The risk is particularly high in irradiated patients within the first 30 months post‐surgery, emphasizing the need for tailored surgical planning and close monitoring.

The free fibula flap (FFF), introduced by Hidalgo^1^ in 1989, is the gold standard for mandibular reconstruction due to its robust vascular supply, sufficient bone length, and versatility for contouring through osteotomies.^2^, ^3^, ^4^ Titanium miniplates secure the fibula to the native mandible, providing stability during osseous integration^5^. However, miniplate removal due to complications remains a significant challenge, with reported rates ranging from 10% to 30%^6^, ^7^. This complication can lead to infection, delayed healing, and the need for additional surgeries, ultimately compromising patient outcomes.

Previous research has identified several potential risk factors, including patient habits like smoking^8^ and adjuvant treatments such as radiotherapy.^9^, ^10^ It is well‐established that radiotherapy impairs wound healing and elevates infection risk following FFF reconstruction. Concurrently, surgical complexity, exemplified by the number of osteotomies, may compromise the flap's vascular supply and the integrity of its soft tissue envelope. Although these detrimental factors are acknowledged, there is no consensus on their relative significance or interactive effects.^11^ Furthermore, the specific impact of surgical complexity on the incidence of miniplate failure has not been consistently established.

This study, therefore, aims to identify the most critical risk factors for miniplate removal in a large group of patients who underwent FFF oromandibular reconstruction. We specifically hypothesized that adjuvant radiotherapy and surgical complexity—defined by the number of osteotomies—would be significant predictors of hardware failure. By analyzing patient characteristics, complication rates, and long‐term outcomes, we seek to provide clearer evidence to guide surgical planning and improve patient monitoring.

## Methods

### Study Design and Population

This retrospective cohort study was conducted at Kaohsiung Veterans General Hospital, a tertiary referral center in Taiwan, analyzing patients who underwent oromandibular reconstruction with FFF fixed by miniplates between January 2015 and December 2023. The study was approved by the Kaohsiung Veterans General Hospital Ethics Committee (KSVGH20‐CT12‐08), with a waiver of informed consent due to its retrospective nature.

Inclusion criteria were (1) age ≥18 years, (2) FFF reconstruction for mandibular defects (oncologic, traumatic, or osteoradionecrotic), and (3) osseous fixation with miniplates and screws. Exclusion criteria included incomplete medical records, alternative fixation methods (eg, reconstruction plates), flap failure, plate removal due to tumor recurrence, or follow‐up <6 months. A total of 184 patients were included.

### Data Collection

Data were extracted from electronic medical records, encompassing patient demographics (age, sex, body mass index), comorbidities (diabetes mellitus, hypertension), and lifestyle factors (smoking, alcohol consumption, betel nut use). Surgical details included fibula bone length, number of osteotomies, and defect type classified using the Schultz system. Information on adjuvant therapies, specifically preoperative or postoperative radiotherapy (66‐70 Gy) and chemotherapy, was also collected. Outcome measures comprised miniplate removal status and associated complications, such as abscess or fistula formation, plate loosening, osteomyelitis, and osteoradionecrosis.

### Surgical Protocol

Titanium miniplates were contoured to the native mandible and pre‐fixed to residual bone segments before resection to preserve geometry. In cases of cortical bone invasion, plates were shaped post‐resection using maxillomandibular fixation and double plating. All patients received conventional titanium miniplates for osseous fixation. No low‐profile plates, locking plates were used during the study period. Plate selection was limited to conventional miniplate configurations (straight vs curved, different lengths) based on defect geometry and surgeon preference, ensuring hardware homogeneity across the cohort. All fibula osteotomies were performed using conventional freehand techniques without virtual surgical planning or computer‐aided guidance. Osteotomy sites were determined intraoperatively based on mandibular defect geometry and surgeon assessment of anatomical requirements.

### Statistical Analysis

Continuous variables (eg, age, BMI) were reported as means ± standard deviations (SD) and compared using independent *t*‐tests or Mann‐Whitney *U* tests for skewed data. Categorical variables (eg, sex, smoking) were expressed as frequencies (%) and analyzed with chi‐square or Fisher's exact tests. Complication rates were compared using chi‐square tests. Kaplan‐Meier analysis assessed time to miniplate removal, stratified by radiotherapy status, with log‐rank testing for differences.

Multivariate logistic regression identified independent risk factors for miniplate removal. Variables with *P* < .10 in univariate analysis were included in the multivariate model. Odds ratios (ORs) with 95% confidence intervals (CIs) were reported. Significance was set at *P* < .05. Analyses were performed using SPSS version 28 (IBM Corp.).

## Results

### Patient Characteristics

Of 184 patients, 154 (83.7%) had no miniplate removal, and 30 (16.3%) required removal due to exposure or complications. Table 1 summarizes baseline characteristics. The mean age was comparable (56.5 ± 10.3 vs 55.8 ± 9.2 years; *P *= .495), as were BMI (24.55 ± 4.20 vs 24.59 ± 3.77; *P* = .475) and sex distribution (94.2% vs 96.7% male; *P* = .504). Smoking was more frequent in the removal group (86.7% vs 70.8%; *P* = .052), approaching significance. No significant differences were observed for alcohol consumption (51.3% vs 60.0%; *P* = .251), betel nut use (60.4% vs 73.3%; *P* = .128), diabetes (18.8% vs 20.0%; *P* = .527), or hypertension (34.4% vs 43.3%; *P* = .233). Radiotherapy was more frequent in the removal group (66.7% vs 21.4%; *P* < .001). The number of osteotomies performed in the fibula bone ranged from 0 to 2 or more, with the distribution shown in Table 2.

**Table 1:** Comparison of Patient Characteristics Based on Miniplate Status

Characteristic	Miniplate not removed (n = 154)	Miniplate removal (n = 30)	*P*‐value
Age (years, mean ± SD)	56.5 ± 10.3	55.8 ± 9.2	.495
Sex (male: female)	144:10:00	29:1	.504
BMI (mean ± SD)	24.55 ± 4.20	24.59 ± 3.77	.475
Smoking, n (%)	109 (70.8)	26 (86.7)	.052
Alcohol consumption, n (%)	79 (51.3)	18 (60.0)	.251
Betel nut use, n (%)	93 (60.4)	22 (73.3)	.128
Diabetes mellitus, n (%)	29 (18.8)	6 (20.0)	.527
Hypertension, n (%)	53 (34.4)	13 (43.3)	.233

Abbreviation: SD, standard deviation.

**Table 2:** Distribution of Osteotomies and Association with Miniplate Removal

Number of osteotomies	Miniplate not removed (n = 154)	Miniplate removed (n = 30)	Total (n = 184)
0 osteotomies	100 (64.9%)	15 (50.0%)	115 (62.5%)
1 osteotomy	49 (31.8%)	13 (43.3%)	62 (33.7%)
2 or more osteotomies	5 (3.2%)	2 (6.7%)	7 (3.8%)

Oral squamous cell carcinoma was the primary diagnosis in 83.2% of cases, followed by osteoradionecrosis (5.6%) and odontogenic tumors (2.3%). Mean mandibular defect length was 95.0 ± 23.12 mm. The mean follow‐up duration was 39.38 months.

### Complications

Table 3 compares complication rates. The removal group had significantly higher rates of abscess/fistula (53.3% vs 5.8%; *P* < .001), plate loosening (30.0% vs 2.6%; *P* < .001), osteomyelitis (43.3% vs 0.6%; *P* < .001), and osteoradionecrosis (23.3% vs 2.6%; *P* < .001). These findings indicate a strong association between miniplate removal and severe postoperative morbidity.

**Table 3:** Comparison of Complications Between Patients With and Without Miniplate Removal Following Oromandibular Reconstruction Using Free Fibula Flap

Complication	Miniplate not removed (n = 154)	Miniplate removed (n = 30)	*P*‐value
Abscess/fistula, n (%)	9 (5.8)	16 (53.3)	<.001
Loosening, n (%)	4 (2.6)	9 (30.0)	<.001
Osteomyelitis, n (%)	1 (0.6)	13 (43.3)	<.001
Osteoradionecrosis, n (%)	4 (2.6)	7 (23.3)	<.001

### Survival Analysis

**Figure 1:**
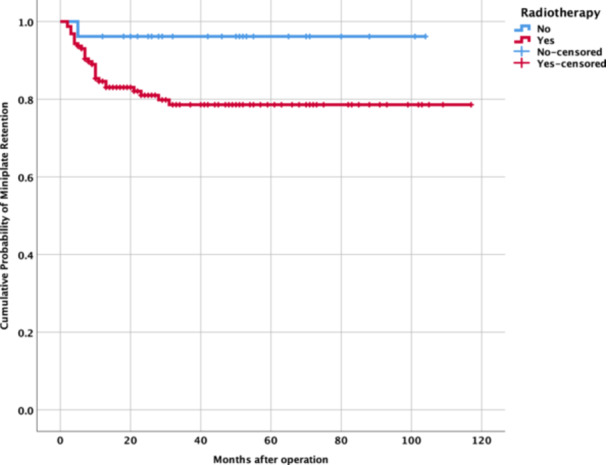
Kaplan‐Meier curves for miniplate retention. Kaplan‐Meier curves depicting miniplate retention over 120 months in oromandibular free fibula flap reconstruction, stratified by radiotherapy (red: radiotherapy; blue: none). Censored data marked by “+”. Log‐rank *P* = .055.

**Figure 2:**
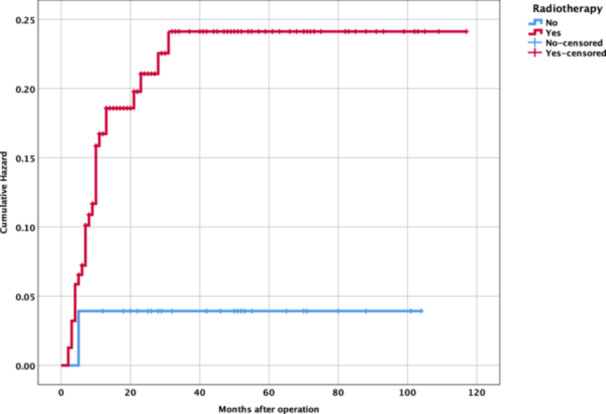
Cumulative hazard of miniplate removal. Cumulative hazard curves depicting miniplate removal risk over 120 months in oromandibular free fibula flap reconstruction, stratified by radiotherapy (red: radiotherapy; blue: none). Censored data marked by “+.”

Kaplan‐Meier analysis (Figures 1 and 2) evaluated time to miniplate removal, stratified by radiotherapy status. The mean time to removal was 34.82 ± 28.84 months (range, 2‐117 months). Due to incomplete event‐time data, definitive survival differences were limited, but log‐rank testing (*P* = 0.055) suggested a trend toward a higher risk of miniplate removal in irradiated patients.

### Risk Factors

Multivariate logistic regression (Table 4) identified 2 independent predictors of miniplate removal: radiotherapy (OR, 9.27; 95% CI, 1.04‐83.04; *P* = .046) and number of osteotomies (OR, 3.77; 95% CI, 1.08‐13.19; *P* = .038). Smoking (OR, 2.46; 95% CI, 0.78‐7.79; *P* = .126) and chemotherapy (OR, 0.61; 95% CI, 0.30‐1.24; *P* = .171) were not significant. Other variables (diabetes, hypertension, bone length, defect type) showed no association (*P* > .05).

**Table 4:** Logistic Regression for Identifying Risk Factors Related to Miniplate Removal

Risk factor	*B* (SE)	Wald	*P*	Odds ratio (95% CI)
Radiotherapy	2.227 (1.118)	3.965	.046*	9.274 (1.036‐83.035)
Chemotherapy	−0.492 (0.359)	1.874	.171	0.611 (0.302‐1.236)
Smoking	0.900 (0.588)	2.347	.126	2.461 (0.778‐7.788)
Fresh case	0.203 (0.458)	0.196	.658	1.225 (0.499‐3.004)
Diabetes mellitus	0.249 (0.564)	0.195	.659	1.283 (0.425‐3.878)
Hypertension	0.143 (0.465)	0.094	.759	1.154 (0.463‐2.873)
Bone length	−0.133 (0.126)	1.119	.29	0.876 (0.685‐1.120)
Numbers of osteotomy	1.327 (0.639)	4.311	.038*	3.769 (1.077‐13.191)
Bone defect (Schultz classification)	−0.061 (0.336)	0.033	.855	0.941 (0.487‐1.816)
Constant	−3.421 (1.582)	4.678	.031	0.033

Abbreviations: CI, confidence interval; SE, standard error.

## Discussion

This study provides a comprehensive analysis of miniplate removal in FFF‐based oromandibular reconstruction, identifying radiotherapy and the number of osteotomies as significant risk factors. The 16.3% removal rate aligns with reported complication rate,^6^, ^7^ highlighting the persistent challenge of hardware‐related complications.

There is a consensus in the literature that radiation therapy, whether administered preoperatively or postoperatively, can have detrimental effects on the soft tissues and bone involved in FFF reconstruction. These negative effects include impaired soft tissue healing, reduced vascularization, and slower bone ossification, which can theoretically increase susceptibility to various postoperative complications. Among the hardware‐related complications, plate removal is consistently reported as a frequent occurrence following FFF surgery for oromandibular reconstruction. Our results demonstrate a strong association between radiotherapy and miniplate removal (OR, 9.27), supporting earlier studies.^12^, ^13^ Radiotherapy induces tissue fibrosis, vascular compromise, and impaired wound healing, increasing susceptibility to infection and osteoradionecrosis.^10^ The wide CI (1.04‐83.04) reflects the small number of irradiated patients in the removal group, warranting cautious interpretation. The Kaplan‐Meier survival curves (Figure 1) demonstrated a higher rate of miniplate removal in the irradiated group compared to the nonirradiated group. Within the first 30 months postoperation, the cumulative probability of miniplate retention in the irradiated group dropped to approximately 60%, while the non‐irradiated group maintained a probability of around 80% throughout the follow‐up period. The log‐rank test was used to compare the survival distributions between the 2 groups, yielding a *P*‐value of .055. This borderline result suggests a trend toward a higher risk of miniplate removal in patients who received radiotherapy, though the difference did not reach statistical significance. To further explore the accumulation of risk over time, we examined the cumulative hazard of miniplate removal for each group (Figure 2). The cumulative hazard in the irradiated group increased rapidly within the first 20 to 30 months, reaching approximately 0.25, and then plateaued, indicating that most of the risk of miniplate removal in this group was concentrated early in the postoperative period. In contrast, the nonirradiated group exhibited a much slower increase in cumulative hazard, reaching only about 0.05 by 120 months, reflecting a lower overall risk of miniplate removal. This early accumulation of risk in the irradiated group aligns with the steeper decline observed in the Kaplan‐Meier survival curve and suggests that radiation‐induced complications, such as infection or osteoradionecrosis, may contribute to early miniplate removal in these patients. These findings further indicate that radiotherapy may be a potential risk factor for miniplate removal, particularly in the early postoperative period. Patients who receive radiotherapy may benefit from closer monitoring during the first 2 to 3 years postsurgery to mitigate complications leading to miniplate removal. Strategies to mitigate radiotherapy‐related risks include optimizing reconstruction timing,^14^, ^15^ using vascularized soft tissue flaps,^16^ and considering alternative fixation methods.^17^, ^18^, ^19^, ^20^, ^21^ Hyperbaric oxygen therapy may also reduce complications in irradiated tissues.^22^

Mandibular reconstruction using the FFF often involves creating multiple segments of the fibula bone through osteotomies to achieve the desired anatomical contour and functional outcome, particularly in the complex three‐dimensional reconstruction of the mandible. In this study, the number of osteotomies (Tables 2 and 4) is a significant predictor (OR, 3.77), likely due to disrupted periosteal blood supply, increased soft tissue tension, and reduced biomechanical stability.^11^, ^23^ The evidence regarding the direct correlation between the number of osteotomies performed during FFF mandibular reconstruction and the risk of plate extrusion or removal is not entirely consistent across the literature. Each osteotomy introduces potential weak points, increasing risks of loosening and exposure. In contrast, some studies have indeed found an association between a greater number of osteotomies and an increased risk of fibula free flap plate removal, other studies have not consistently observed this correlation. This suggests that the relationship might not be straightforward and could be influenced by other confounding factors. Surgeons should strive to balance the need for anatomical accuracy with the goal of minimizing the number of osteotomies, especially in patients who present with other known risk factors for plate extrusion, such as a history of smoking or prior radiation therapy. The selection of the appropriate fixation method is also critical and should be tailored to the complexity of the reconstruction and the individual patient's characteristics. Computer‐aided surgical planning and piezoelectric surgery may minimize osteotomy‐related complications^24^, ^25^ by enhancing precision and preserving vascularity.

Clinical observations indicate that the exposure of miniplates following FFF oromandibular reconstruction can lead to a cascade of severe complications. Patients experiencing exposure and subsequent removal of hardware have demonstrated significantly elevated rates of specific morbidities. Notably, debridement was required in an overwhelming majority of these cases (96.7%). This near‐universal need for surgical debridement suggests that miniplate exposure is not a minor event but rather a critical trigger necessitating active intervention to address the compromised tissues. Consequently, the hardware itself may need to be removed either concurrently with or following debridement to ensure the complete eradication of infection and to promote optimal tissue recovery. Furthermore, substantial rates of osteomyelitis (43.3%) and osteoradionecrosis (23.3%) were observed in this study. These findings suggest a potential pathway where the exposure of the hardware compromises the soft tissue barrier, facilitating bacterial entry and increasing the risk of infection that can extend to the bone, potentially leading to osteomyelitis.^26^ In patients with a history of radiation therapy, the exposure might also exacerbate or contribute to the development of osteoradionecrosis due to the already compromised healing capacity of the irradiated tissues. These severe complication rates underscore the cascading morbidity associated with miniplate exposure, emphasizing the critical need for early detection and aggressive management strategies. Preventive measures, particularly those focused on achieving and maintaining robust soft tissue coverage over the miniplates, are therefore of paramount importance in mitigating these risks.

Despite high success rates, complications such as flap failure, surgical site infections, and hardware‐related issues (eg, plate extrusion or exposure) can occur. Lifestyle habits, including smoking and alcohol consumption, and comorbidities like diabetes and poor overall health status, are hypothesized to influence these outcomes. Although smoking demonstrated borderline significance (*P* = .052), consistent with its known role in impairing wound healing, it did not remain a significant factor in the multivariate analysis.^27^, ^28^ This may reflect high baseline smoking prevalence (70.8%) in this cohort. The combined influence of DM, hypertension, and BMI on the overall success and complication rates of free fibula oromandibular reconstruction, particularly concerning hardware‐related issues, is intricate and likely involves a combination of factors. As previously discussed, DM has been linked to an increased risk of overall surgical complications and flap failure in head and neck free flap surgery. While a direct link to hardware removal is not always evident in multivariate analyses, the heightened risk of surgical site infections in diabetic patients could certainly contribute to this specific complication.^26^

### Clinical Implications

These findings advocate for tailored strategies in high‐risk patients. Pre‐radiotherapy soft tissue augmentation, alternative fixation methods (eg, locking plates, CAD/CAM reconstruction plates, hybrid CAD/CAM miniplate systems^29^), and smoking cessation programs may reduce hardware removal risk. Multidisciplinary planning is essential to balance oncologic and reconstructive goals.

### Strengths and Limitations

Strengths include a large sample size (N = 184), detailed data collection, and robust statistical methods. Limitations include the retrospective design, single‐center setting, and incomplete Kaplan‐Meier data, which limited survival analysis. The small miniplate removal group (n = 30) may have reduced statistical power for some variables. This study has several limitations. First, as a retrospective analysis, the selection of flaps was based on surgeon preference rather than randomization, which may introduce selection bias and limit control for confounding variables. Second, the study population included both oral cancer patients and those with benign conditions who did not require neoadjuvant or adjuvant therapy, potentially introducing heterogeneity in treatment outcomes. An important limitation of this study is the inability to distinguish between the effects of preoperative versus postoperative radiotherapy on miniplate removal outcomes. Due to the complex clinical scenarios involving tumor recurrence and multiple surgical interventions, along with the small sample size of patients experiencing miniplate removal (n = 30), we could not stratify outcomes by radiotherapy timing. Future prospective studies with larger sample sizes should specifically examine whether the timing of radiotherapy (preoperative vs. postoperative) differentially affects hardware‐related complications in free flap reconstruction. Another important limitation of this study is its focus on miniplate fixation, which may limit generalizability to centers primarily using reconstruction plates. While the biological mechanisms underlying our identified risk factors (radiotherapy‐induced tissue compromise and osteotomy‐related complications) likely apply across fixation methods, the specific complication profiles and management strategies may differ. Future multicenter studies comparing outcomes across different fixation systems would help validate these findings in diverse practice settings.

### Future Directions

Prospective, multicenter studies are needed to validate these findings and explore novel fixation strategies, such as custom plates. Biomechanical analyses and cost‐effectiveness studies could further optimize reconstructive approaches. Long‐term functional outcomes (eg, mastication, speech) warrant investigation.

## Conclusions

Miniplate removal in oromandibular reconstruction with FFFs are significantly associated with preoperative or postoperative radiotherapy and an increased number of osteotomies. These complications, observed in 16.3% of patients, underscore the importance of individualized surgical strategies and vigilant postoperative monitoring, particularly within the first 30 months for irradiated patients. Multidisciplinary approaches, incorporating optimized reconstruction timing, advanced fixation techniques, and preventive measures like smoking cessation, are critical to minimizing hardware‐related complications. Future prospective, multicenter studies and innovations in fixation methods are essential to improving patient outcomes in mandibular reconstruction.

## Author Contributions

The contributions of each author to the study titled “Risks of Miniplate Removal in Fibula Free Flap Oromandibular Reconstruction” are detailed below in accordance with standard academic authorship guidelines. Each author has reviewed and approved the final manuscript and agrees to be accountable for all aspects of the work.

**Kuo‐Chung Yang**, performed surgical procedures, contributed to the conceptualization of the study, participated in data collection, and provided critical suggestions for study design and manuscript revisions. **Wen‐Chung Liu**, served as the corresponding author, performed surgical procedures, contributed to the conceptualization of the study, participated in data collection, and was responsible for drafting and revising the manuscript.

## Disclosures

### Competing interests

None.

### Funding source

None.
